# Professional Quality of Life and Mental Health Outcomes among Health Care Workers Exposed to Sars-Cov-2 (Covid-19)

**DOI:** 10.3390/ijerph17176180

**Published:** 2020-08-26

**Authors:** Rodolfo Buselli, Martina Corsi, Sigrid Baldanzi, Martina Chiumiento, Elena Del Lupo, Valerio Dell’Oste, Carlo Antonio Bertelloni, Gabriele Massimetti, Liliana Dell’Osso, Alfonso Cristaudo, Claudia Carmassi

**Affiliations:** 1 Occupational Health Department, Azienda Ospedaliero-Universitaria Pisana, Via Paradisa 2, 56124 Cisanello (Pisa), Italy; r.buselli@gmail.com (R.B.); sigridbaldanzi@gmail.com (S.B.); martina.chiumiento@gmail.com (M.C.); psico.ele@gmail.com (E.D.L.); alfonso.cristaudo@med.unipi.it (A.C.); 2 Psychiatric Clinic, Department of Clinical and Experimental Medicine, University of Pisa, Pisa, Via Roma 67, 56100 Pisa, Italy; valerio.delloste@gmail.com (V.D.); carlo.ab@hotmail.it (C.A.B.); gabriele.massimetti@med.unipi.it (G.M.); liliana.dellosso@med.unipi.it (L.D.); ccarmassi@gmail.com (C.C.)

**Keywords:** COVID-19, healthcare workers (HCWs), professional quality of life (ProQOL-5), compassion satisfaction (CS), burnout, secondary traumatization (ST)

## Abstract

The novel coronavirus disease 2019 (COVID-19) is a global pandemic spreading worldwide, and Italy represented the first European country involved. Healthcare workers (HCWs) facing COVID-19 pandemic represented an at-risk population for new psychosocial COVID-19 strain and consequent mental health symptoms. The aim of the present study was to identify the possible impact of working contextual and personal variables (age, gender, working position, years of experience, proximity to infected patients) on professional quality of life, represented by compassion satisfaction (CS), burnout, and secondary traumatization (ST), in HCWs facing COVID-19 emergency. Further, two multivariable linear regression analyses were fitted to explore the association of mental health selected outcomes, anxiety and depression, with some personal and working characteristics that are COVID-19-related. A sample of 265 HCWs of a major university hospital in central Italy was consecutively recruited at the outpatient service of the Occupational Health Department during the acute phase of COVID-19 pandemic. HCWs were assessed by Professional Quality of Life-5 (ProQOL-5), the Nine-Item Patient Health Questionnaire (PHQ-9), and the Seven-Item Generalized Anxiety Disorder scale (GAD-7) to evaluate, respectively, CS, burnout, ST, and symptoms of depression and anxiety. Females showed higher ST than males, while frontline staff and healthcare assistants reported higher CS rather than second-line staff and physicians, respectively. Burnout and ST, besides some work or personal variables, were associated to depressive or anxiety scores. The COVID-19 pandemic represents a new working challenge for HCWs and intervention strategies to prevent burnout and ST to reduce the risk of adverse mental health outcomes are needed.

## 1. Introduction

### 1.1. Literature Background

The pandemic of the severe acute respiratory coronavirus 2 (SARS-COV-2) and its associated disease, named coronavirus disease 19 (COVID-19), emerged in Wuhan, China, at the end of December 2019, then spread to the entire country, and attracted enormous concern from around the world [1]. Up to 1 July 2020, more than 10 million confirmed cases of COVID-19 have been diagnosed worldwide, with 506,064 deaths (WHO: Coronavirus disease (COVID-19) Situation Report 163 1 July 2020) [2,3].

According to literature from SARS or Ebola epidemics, the onset of a sudden and immediately life-threatening illness could lead to exceptional levels of pressure on healthcare workers (HCWs). Increased workload, physical pressure, isolation and loss of social support, inadequate protective measures, professional viral transmission, and unprecedented ethical concerns on the rationing of care may have important consequences on their personal physical and mental well-being [4,5,6,7].

The actual outbreak has markedly changed the working scenario and job demands. Current literature reports a proportion of infected hospital staff of 3.8%, mainly due to early non-protected contact with infected patients. HCWs need to wear heavy personal protective equipment (PPE), making it difficult to carry out medical procedures. These factors, together with the fear of being contagious and infecting others, could increase the risk of psychological consequences. Taking care of patients, for the first time, mixed with HCWs concern for their one’s health, uncertainty, emotional difficulties, and stigmatization. HCWs reported conflicting thoughts and difficulties about balancing their professional roles and their familiar duties. They referred to the struggles of weighing responsibility and a sense of guilt [7,8,9].

Current literature describes that HCWs on the front line have proven to be more at risk of developing psychological symptoms and mental health disorders [10]. Frontline workers are, in fact, directly responsible in the caring process of patients with COVID-19 and have to face peculiar psychosocial risk factors such as the depletion of PPE, lack of specific guidelines of treatment, and feelings of being inadequately supported, which may all contribute to their mental burden [10,11].

Some studies reported that those HCWs who feared infection of their close one’s, reported experiencing high levels of stress, anxiety, and depression symptoms, which could have long-term psychological implications [8,11]. A multicenter cross-sectional study on more than 1000 Chinese HCWs evidenced percentages of distress, depression, anxiety, and insomnia of 71.5%, 50.4%, 44.6%, and 34.0%, respectively [11,12]. The best evidence based on a systematic review and meta-analyses of 13 studies and a total of 33,062 subjects confirmed that a high number of HCWs are experiencing significant levels of anxiety, depression, and insomnia during the COVID-19 outbreak. The prevalence rates of anxiety and depression were 23.2% and 22.8%, respectively. It appeared that a high proportion of HCWs reported mild symptoms both for depression and anxiety, while on the contrary, moderate and severe symptoms were less common. [3,7,12].

Nevertheless, in battling SARS, psychological distress among HCWs appeared gradually. In particular, fear and anxiety appeared immediately and decreased in the early stages of the outbreak, but depression, psychophysiological symptoms, and post-traumatic stress symptoms appeared in a second moment and lasted for a long time, leading to a deep impact [13,14].

At the same time, beyond the new burden of psychosocial risk factors, COVID-19 brought also positive elements that should be analyzed. The public response toward HCWs has been globally heart-warming as never before. Numerous testimonies report worldwide expressions of gratitude and closeness to HCWs. The pandemic has put HCWs in the spotlight, and for some, it has been an important positive reinforcement. Others have hypothesized that these peculiar elements related to COVID-19 have somehow buffered the burnout effects, at least in the first phase, giving a profound sense of self-efficacy [15,16].

As previously described, the pandemic has exposed HCWs to several elements derived from completely new work-related circumstances that contribute to a new complex work environment [1]. In the current healthcare context, signs of occupational stress are an important public health concern [4,5,6,7]. Repeated exposure to unpredictable challenges in practice may cause, on one hand, symptoms of anxiety, exhaustion, and stress described in working context as compassion fatigue (CF, burnout, and ST) but on the other hand public opinion on HCWs improved and everyone gathered around them in a way without historical precedents [1,17,18,19]. This second element can be named CS, which is described as the satisfaction experienced by HCWs when performing their work properly, which also includes satisfaction with their relationship with colleagues and the sense that the work they perform is of social value. Unsurprisingly, the balance between CS and CF determines the level of professional quality of life [17,20,21].

Certain work and personal-related factors may influence quality of life among HCWs. Specifically, CF and CS have already been reported to be related to the healthcare setting and the work environment. Nevertheless, previous studies analyzing these factors do not determine exactly what socio-demographic and work-related variables may influence professional quality of life and HCWs global health [17,20,21,22,23].

What is quite evident is that COVID-19 demonstrates a dramatic gap in the current scientific literature regarding these dimensions and the related mental health of HCWs [24,25,26]. Surprisingly, while Italy has been deeply struck by the pandemic, only two studies provide evidences about the COVID-19 subjective mental experience; Ramaci et al. (2020) report some findings about the effect of social stigma on workers’ psychological well-being to be further ascertained [9], and Magnavita et al. [27] show that frequency of anxiety and depression disorders in the population examined were not higher than that commonly recorded during the scheduled checks previous to the epidemic.

Accordingly, it is important for occupational teams to study HCWs health dimensions with both a more detailed characterization of clinical endpoints about baseline workers’ condition before to COVID-19 outbreak and cross-sectional and longitudinal designs.

The scientific community has called for high-quality evidence on the mental health effects of the COVID-19 pandemic across the whole population and vulnerable groups such as HCWs, which has since confirmed that data on this challenging issue are still limited [28]. In this context, it is therefore an essential contribution to literature in order to better understand the effects of COVID-19 pandemic on HCWs’ outcomes.

In light of what has emerged so far, we believe that the pandemic has brought changes in the HCWs working framework, which seems to be acting both positively and negatively on the psychological equilibrium of HCWs. These theories arise from the literature and from our own clinical experience, since we are a multidisciplinary team responsible for the well-being of hospital HCWs and therefore are in close contact with their psychic feedback, especially in the context of the first Italian emergency phase of the pandemic (Azienza OspedalieroUniversitaria Pisana *psicocovid19 protocol*) [8].

In particular, it is fundamental to analyze whether and how the new COVID-19-related working burden is potentially capable of producing changes to the quality of professional life of HCWs.

### 1.2. Study Aim and Hypotheses

The main aim of the present study was to identify the contribution of personal and working contextual variables (gender, working position, years of experience, proximity to infected patients (front-line and second-line workers)) on professional life dimensions (satisfaction, burnout, and secondary traumatization). A further aim was to analyze the possible impact of these dimensions on HCWs’ mental health.

**Figure 1:**
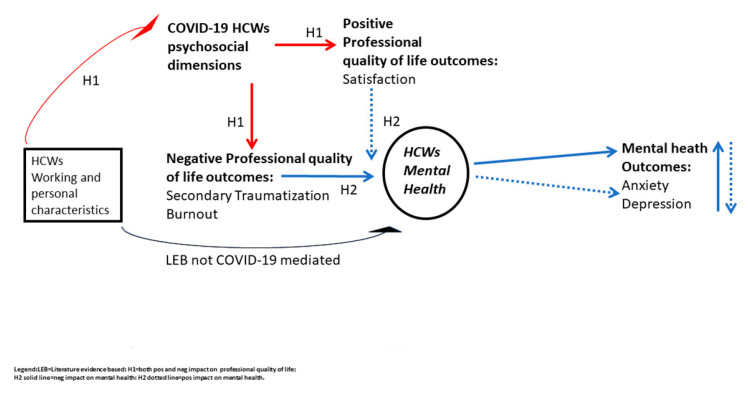
Overall theoretical model of study hypothesis (H1 and H2).

As already mentioned, after considering recent evidence, it is possible to hypothesize both a positive and negative influence of COVID-19 working and personal variables on professional quality-of-life dimensions. We hypothesize a double and opposite influence of the individual characteristics but not the single term of influence for each factor since COVID-19 is an unpredictable new event. Further, it is possible to hypothesize a consequent and related impact of professional quality-of-life dimensions on mental health outcomes. The same independent variables (HCW characteristics) obviously present a direct impact on HCWs’ mental health, which is completely independent of the pandemic, as highly demonstrated by the literature on HCWs through years [28,29,30]. (See Figure 1 for theoretical model of study hypothesis).

## 2. Materials and Methods

### 2.1. Study Sample and Procedures

**Figure 2:**
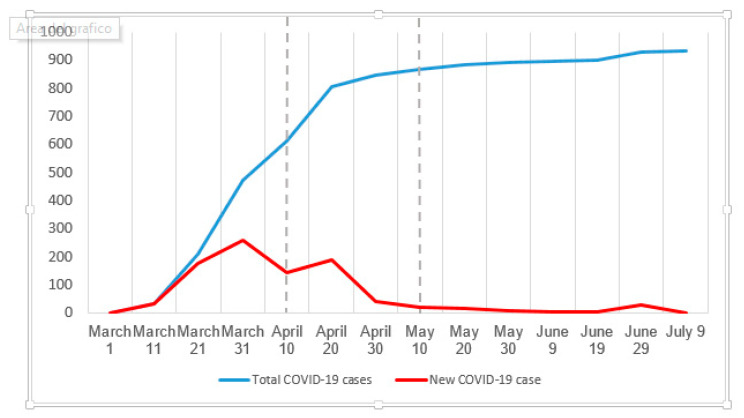
Case count (unit) of COVID-19 infection in Pisa (Italy) before, during, and after the study period.

A cross-sectional study was designed and conducted in accordance with the STROBE (STrengthening the Reporting of OBservational studies in Epidemiologyrecommendation for observational studies and it was carried out between 1 April and 1 May 2020 at the outpatient service of the Occupational Health Department of a major university hospital in central Italy (Azienda Ospedaliero-Universitaria Pisana, AOUP, Pisa, Italy). As showed in Figure 2, the study was conducted during the second part of the height of the pandemic in Pisa, when HCWs experienced the greatest work-related stress burden. The total sample included 265 HCWs employed at the AOUP during the Italian COVID-19 pandemic phase 1.

Some working contextual and personal variables were collected—age, gender, working role (nurse, physician, health care assistant), intensive care unit (ICU) working or not, frontline working or not, time spent on hospital working duty <1 year (new employed) or ≥1 year.

We decided to give priority to working variables than to personal in order to optimize the possible drops since HCWs were not always willing to allow their time or to give too much personal information, especially in a such a particular period. Age and gender are immediately available and already present literature evidence of their impact on the quality of professional life, but with few new observations in the COVID-19 context [17,31].

Participants were informed about all main aspects of the research and of their right to refuse to take part or to withdraw consent to participation at any moment. They then confirmed that they fully understood the instructions and verbally accepted participation in the study. The study was conducted in accordance with the 1964 Helsinki Declaration and was approved by the Ethics Committee of Area Vasta Nord-Ovest Toscana (Italy).

### 2.2. Measures

The whole sample was investigated by means of the Professional Quality Of Life Scale version 5 (ProQOL-5) [32] to assess compassion satisfaction (CS), burnout, and secondary traumatization (ST) related to their HCWs role; the Generalized Anxiety Disorder Seven-Item (GAD-7) [33] to explore anxiety symptoms; and the Patient Health Questionnaire-9 (PHQ-9) [34] to examine depressive symptoms. Sociodemographic characteristics were also gathered through a specific datasheet reporting information on the COVID-19 pandemic.

The ProQOL-5 by Stamm [32] was used to analyze the professional quality of life of the study sample. ProQOL integrates two aspects, namely, compassion satisfaction (CS) and compassion fatigue (CF). CF has two factors: burnout (e.g., exhaustion, frustration, anger and depression) and secondary traumatization (ST). ProQOL is a 30-item Likert scale ranging from 1 (never) to 5 (very often). The category raw scores may range from 10 to 50; score ranges are also available for each category. The CS scale had Cronbach’s alpha of 0.88, whereas the Burnout and ST scales had Cronbach’s alphas of 0.75 and 0.81, respectively [32].

The PHQ-9 [34] is a nine-item tool of depression that reflects the criteria for major depressive disorder from the Diagnostic and Statistical Manual of Mental Disorders (DSM). Study sample rate the frequency of experiencing a specific symptom over the previous two weeks. Two of the items on the PHQ-9 refer to somatic symptoms—fatigue and sleep disturbance— and the other items refer to non-somatic symptoms. Items are rated using a four-point scale ranging from 0 (“Not at all”) to 3 (“Nearly every day”).

The GAD-7 [33] is a tool developed for clinical applications and uses the DSM clinical diagnostic criteria for generalized anxiety disorder. Seven items refer to symptoms of anxiousness, worry, fear, and irritability occurring over the previous two weeks and are rated on a frequency scale from 0 (“Not at all”) to 3 (“Nearly Every Day”).

### 2.3. Statistical Analyses

All statistical analyses were performed using the Statistical Package for Social Science, version 25.0 (IBM Corp. Released 2017. IBM SPSS Statistics for Windows, Version 25.0. Armonk, NY: IBM Corp.Continuous variables were reported as mean ± standard deviation (SD), whereas categorical variables were reported as percentages. All tests were two-tailed, and a *p* value < 0.05 was considered statistically significant.

To compare the three ProQOL subscales, the PHQ-9 and the GAD-7 scores among dichotomic working and personal characteristics variables the non-parametric Mann–Whitney U-test was used. To compare the three ProQOL subscales scores, the PHQ-9 and the GAD-7 scores among the three professional groups the non-parametric Kruskar-Wallis test, followed by Dunn test for pairwise comparisons, was performed. Non-parametric analyses were utilized because ProQOL, GAD-7, and PHQ-9 scores did not present a normal distribution. In descriptive analysis, their median, in fact, was not included in the 95% confidence interval of the mean scores. The abnormal distribution of the variables was corroborated by Q–Q plots and Kolmogorov–Smirnova and Shapiro–Wilk tests. Choen’s d was calculated in order to provide effect size estimates. In the light of the observational nature of the study and the difference among the personal and work-related variables involved in the comparison analyses, we did not consider it necessary to correct for multiple testing.

Two multiple linear regression models were utilized to study the strongest predictor of PHQ-9 and GAD-7 (dependent variables) among gender, frontline staff, ICU staff, hospital workers for more or less than one year, and the three ProQOL subscales scores (as independent variables).

## 3. Results

The study included a total sample of 265 HCWs, 84 (31.7%) males and 181 females (68.9%), employed in COVID-19 hospital wards. The mean age in the total sample was 40.4 ± 11.2 (min 19, max 63) years. Furthermore, 85 (32.1%) were medical doctors, 133 (50.2%) were nurses, and 47 (17.7%) were healthcare assistants. Fifty-one (19.2%) HCWs had spent less than one year on hospital duty. During the COVID-19 pandemic 117 (44.2%) subjects had a first line activity, while 78 (29.4%) worked in an Intensive Care Unit. The PHQ-9 mean score was 4.5 ± 6.4, while the GAD-7 mean score was 4.2 ± 4.6.

In relation to the ProQOL-5 subscales scores, the CS mean score was 38.2 ± 7.0 (min 9, max 76), the Burnout mean score was 19.8 ± 5.0 (min 7, max 54), and the ST mean score was 18.0 ± 5.6 (minimum 7, maximum 39). Comparisons of the three ProQOL-5 subscales scores among working and personal characteristics variables are shown in Table 1.

In a linear regression model, considering sociodemographic variables (age, gender, physician role, first line activity, working in an ICU) and the three ProQOL-5 subscales scores as independent variables, and the PHQ-9 total scores as the dependent variables, the burnout (b = 0.4 (SE = 0.10), *p* < 0.001) and Secondary Traumatic Stress (b = 0.2 (SE = 0.09), *p* = 0.007) scores presented a significant positive association with the PHQ-9 scores. See Table 2.

In a similar linear regression model, considering sociodemographic variables (age, gender, physician role, first line activity, working in an ICU) and the three ProQOL-5 subscales scores as independent variables, and the GAD-7 total scores as the dependent variables, burnout (b = 0.20 (SE = 0.06), *p* = 0.001) and secondary traumatic stress (b = 0.42 (SE = 0.05), *p* < 0.001)scores, beside first line activity [b = 1.76 (SE = 0.65), *p* = 0.008] and working in an ICU (b = −2.29 (SE = 0.71), *p* = 0.001) presented a significant association with the GAD-7 scores. (See Table 3.)

## 4. Discussion

In this hypothesis-testing study, supported by a hospital sample of HCWs tested in close temporal proximity to the period of Italian lockdown, we examined the role of some personal and working characteristics on professional quality of life dimensions and the subsequent impact on anxiety and depression. Overall, the results of this study seem to provide evidence in line with the reported literature and with the proposed theoretical model (Figure 1).

What emerged, in fact, is that HCWs exposed to COVID-19 psychosocial new challenges (Italian Hospital phase 1) experienced both negative and positive psychological outcomes at the same time.

The results show that working in the front line or being a physician positively impacts CS. We can argue that physicians in general and frontline staff may have felt more gratification and a deeper sense of personal success perceiving the direct effects of their treatments on patients affected by COVID-19 [16]. At the same time, being in front line and working in an ICU demonstrated to be a potential risk factor for anxiety but not for depressive symptoms.

Many previous studies have shown that HCWs, especially nurses, with high level of stress were more prone to develop anxiety, frustration, depression, and other psychological disorders [35]. Magnavita et al. [27] showed that, despite the fact that the overall prevalence of common mental disorders in the entire HCWs population is not higher than that recorded during periodic medical visits to the workplace, in exposed workers, the risk of both anxiety and depression was double compared to controls.

The absence of significant levels of CF through the groups is in line with previous findings [27] and could be due to different factors: (1) CS may have balanced CF in this particular pandemic situation, (2) the analysis were done probably too early to see the real effects of the pandemic on burnout [13,14], (3) information about HCWs’ mental condition previous to the outbreak were not available.

Consistently, various authors have provided the rationale of positive growth subsequently a traumatic event and life crisis [36]. It is also possible that physicians with respect to others health care professionals may have felt closer to the key decision makers and have had access to more timely and accurate information [37]. They probably perceived more public support and less the weight of suffering [8]. Some Chinese authors reported corroborating findings. They highlighted how burnout frequency was lower among HCWs working on the COVID-19 frontline compared to those working their usual wards [16].

As already mentioned, the findings indicate also that women reported more statistically significant higher scores at ST than men and this evidence is in line with previous gender literature and recent research on pandemics in Italy [27]. There is general consensus that women have a two to three times higher risk of developing post traumatic stress symptoms as compared to men [38,39]. Women with trauma exposure exhibit greater sensitivity and lower tolerance to negative emotions than men. Consistently, women appear to have a more sensitized hypothalamus–pituitary axis [38,39,40,41,42].

ST is, in fact, similar to post-traumatic stress but results from knowledge of traumatic events suffered by others and the consequent stress. It is the emotional residue or strain of exposure to working with those suffering from the consequences of traumatic events. Differently from burnout, it has a more rapid onset while burnout emerges over time [43]. The fact that burnout emerges over time is probably another reason why we have not found alarming levels of burnout except for a trend emerged in the new hires focusing on the need for longitudinal studies.

Others noteworthy findings, in line with the study hypothesis, are the statistically significant negative impact of ST on HCWs mental health both in term of anxiety than depression. This is noteworthy as it makes clear that the event itself is not so much a risk factor but the perception of the event as traumatic [37,38,39]. Evidence that has emerged from the current study should be interpreted in light of some limitations. First, the small sample size cannot be said to have epidemiological value, and difficulties in collecting data about HCWs’ mental condition previous to the outbreak have been limiting factors to draw a clear comparison with the clinical measures collected in our study. To generalize our considerations and have an adequate view of the phenomena we have to wait for longitudinal outcomes. Second limitation is that self-reported tools of psychological impact, anxiety, depression and stress may not always be aligned with assessment by mental health professionals. Third, it is noteworthy that some manifestations of burnout and secondary traumatization partially overlap with depression or anxiety symptoms, as can be realized observing the items of the ProQOL, PHQ-9, and GAD-7 questionnaires. Further, this study was unable to distinguish the association of symptoms with being an HCW or simply experiencing the pandemics since we used a convenient sample to carry out data collection in timely proximity to the COVID-19 outbreak. A next step will be to find a fittable control group to strengthen and better understand the findings.

## 5. Conclusions

In public health emergencies, the psychological burden of HCWs should be focused. Immediate interventions are essential in order to enhance psychological resilience and strengthen the healthcare systems’ capacity. This first phase of a larger research project supports the need to conduct further qualitative research to further explore the sources of HCWs’ satisfaction or dissatisfaction in their challenging role. The future goal would be to evaluate suitable endpoints and develop intervention strategies to reduce the risk of adverse mental health outcomes.

## Figures and Tables

**Table 1:** Professional Quality of Life Scale version 5 (ProQOL-5) subscales and Patient Health Questionnaire-9 (PHQ-9) and Generalized Anxiety Disorder Seven-Item (GAD-7) scores (mean values ± SD) in the total sample (N = 265) and divided by healthcare workers’ (HCWs’) working and personal characteristics.

	N (%)	Compassion Satifaction (Mean ± SD)	*p*	Burnout (Mean ± SD)	*p*	Secondary Traumatic Stress (Mean ± SD)	*p*	PHQ-9 (Mean ± SD)	*p*	GAD-7 (Mean ± SD)	*p*
Total sample	265 (100.0)	38.2 ± 7.0	-	19.8 ± 5.0	-	18.0 ± 5.6	-	4.5 ± 6.4	-	4.2 ± 4.6	-
Male	84 (31.7)	37.3 ± 6.4	0.095 (Choen’s d = −0.19)	19.2 ± 4.1	0.519	16.8 ± 4.7	0.041 (Choen’s d = −0.34)	4.0 ± 5.8	0.412	3.3 ± 4.0	0.020 (Choen’s d = −030)
Female	181 (68.9)	38.6 ± 7.3		20.1 ± 5.3		18.6 ± 5.9		4.8 ± 6.7		4.7 ± 4.8	
ICU staff	78 (29.4)	39.3 ± 5.7	0.084	19.9 ± 5.0	0.586	18.0 ± 6.3	0.535	4.9 ± 6.5	0.088	4.5 ± 4.6	0.073
no-ICU staff	187 (70.6)	37.8 ± 7.5		19.7 ± 4.8		18.0 ± 5.3		3.7 ± 6.2		3.7 ± 4.5	
First line staff	117 (44.2)	39.4 ± 5.5	0.008 (Choen’s d = 0.31)	19.8 ± 4.9	0.598	18.3 ± 6.1	0.955	4.7 ± 5.9	0.245	4.1 ± 3.9	0.486
no-First line staff	148 (55.8)	37.3 ± 7.9		19.8 ± 5.0		17.9 ± 5.2		4.4 ± 7.0		4.5 ± 5.3	
Hospital duty time <1 year	51 (19.2)	39.3 ± 6.4	0.205	18.6 ± 3.9	0.084	17.2 ± 6.0	0.103	4.9 ± 6.4	0.13 (Choen’s d = 0.30)	4.5 ± 4.7	0.014 (Choen’s d = 0.35)
Hospital duty time ≥1 year	214 (80.8)	38.0 ± 7.2		20.1 ± 5.1		18.2 ± 5.5		3.0 ± 6.4		3.0 ± 3.8	
Physicians	85 (32.1)	37.1 ± 5.6	0.021 *	20.2 ± 4.4	0.062	18.0 ± 5.7	0.208	5.4 ± 6.2	0.083	4.4 ± 3.8	0.087
Nurses	133 (50.2)	38.4 ± 6.8		19.9 ± 4.7		18.5 ± 5.8		4.4 ± 6.7		4.56 ± 5.23	
Healthcare assistants	47 (17.7)	40.0 ± 9.4		18.9 ± 6.6		16.7 ± 4.9		3.4 ± 5.8		3.1 ± 3.6	

* In post-hoc pair-wise comparison (Dunn test): physicians versus health care assistants *p* = 0.030.

**Table 2:** Linear regression model: sociodemographic variables and ProQOL-5 subscales scores as predictive variables associated with PHQ-9 score in the total sample (N = 265).

Predictive Factors	b (S.E.)	β	CI_95%_	*p*
Age	−0.01 (0.04)	−0.02	−0.078–0.060	0.794
Gender	−0.42 (0.83)	−0.03	−2.045–1.210	0.614
Physician role	0.94 (0.84)	−0.07	−0.722–2.600	0.266
First line activity	1.36 (1.14)	0.10	−0.885–3.597	0.234
ICU working	−2.08 (1.23)	−0.15	−4.506–0.351	0.093
Compassion Satisfaction	−0.03 (0.05)	−0.04	−0.138–0.074	0.557
Burnout	0.39 (0.10)	0.30	0.190–0.589	<0.001
Secondary Traumatization	0.24 (0.09)	0.21	0.067–0.421	0.007
k	−5.50 (3.26)	-	−11.930–0.924	0.093

b = unstandardized regression coefficients; β = standardized regression coefficients; CI_95%_ = confidence interval at 95%; k = constant; R^2^ = 0.239; R^2^ corrected = 0.212.

**Table 3:** Linear regression model: sociodemographic variables and ProQOL-5 subscales scores as predictive variables associated with GAD-7 score in the total sample (N = 265).

Predictive Factors	b (S.E.)	β	CI_95%_	*p*
Age	−0.03 (0.02)	−0.07	−0.070–0.009	0.129
Gender	0.15 (0.48)	0.02	−0.789–1.084	0.757
Physician role	0.12 (0.49)	0.01	−0.836–1.075	0.806
First line activity	1.76 (0.65)	0.19	0.470–3.048	0.008
ICU working	−2.29 (0.71)	−0.23	−3.683–0.889	0.001
Compassion Satisfaction	−0.00 (0.03)	−0.00	−0.064–0.058	0.933
Burnout	0.20 (0.06)	0.22	0.085–0.314	0.001
Secondary Traumatization	0.42 (0.05)	0.52	0.319–0.522	<0.001
k	−6.24 (1.88)	-	−9.938–−2.544	0.001

b = unstandardized regression coefficients; β = standardized regression coefficients; CI_95%_ = confidence interval at 95%; k = constant; R^2^ = 0.497; R^2^ corrected = 0.480.
